# A first generation BAC-based physical map of the channel catfish genome

**DOI:** 10.1186/1471-2164-8-40

**Published:** 2007-02-06

**Authors:** Sylvie M-A Quiniou, Geoffrey C Waldbieser, Mary V Duke

**Affiliations:** 1 USDA-ARS/CGRU, 141 Experiment Station Rd, Stoneville, MS 38776, USA

## Abstract

**Background:**

Channel catfish, *Ictalurus punctatus*, is the leading species in North American aquaculture. Genetic improvement of catfish is performed through selective breeding, and genomic tools will help improve selection efficiency. A physical map is needed to integrate the genetic map with the karyotype and to support fine mapping of phenotypic trait alleles such as Quantitative Trait Loci (QTL) and the effective positional cloning of genes.

**Results:**

A genome-wide physical map of the channel catfish was constructed by High-Information-Content Fingerprinting (HICF) of 46,548 Bacterial Artificial Chromosomes (BAC) clones using the SNaPshot technique. The clones were assembled into contigs with FPC software. The resulting assembly contained 1,782 contigs and covered an estimated physical length of 0.93 Gb. The validity of the assembly was demonstrated by 1) anchoring 19 of the largest contigs to the microsatellite linkage map 2) comparing the assembly of a multi-gene family to Restriction Fragment Length Polymorphism (RFLP) patterns seen in Southern blots, and 3) contig sequencing.

**Conclusion:**

This is the first physical map for channel catfish. The HICF technique allowed the project to be finished with a limited amount of human resource in a high throughput manner. This physical map will greatly facilitate the detailed study of many different genomic regions in channel catfish, and the positional cloning of genes controlling economically important production traits.

## Background

Channel catfish production is now the leading aquaculture species in the U.S., with 600 millions pounds processed annually [1]. Consequently, selective breeding of catfish broodstock is ongoing in order to improve the genetic potential of the species for commercial production. To support selective breeding research, molecular tools are being developed to help researchers characterize the catfish genome, which consists of 28 pairs of autosomes and one pair of sex chromosomes, and identify genomic regions that control important production traits. However, a considerable amount of available data has not been organized within the structural framework of catfish chromosomes. Briefly, framework genetic linkage maps have been produced based on microsatellite loci [2] and Amplified Fragment Length Polymorphism (AFLP) loci [3]. Channel catfish Expressed Sequence Tags (EST) have been identified from several tissues [4,5] and clustered and annotated in the Gene Index Project [6]. A new EST project is underway through the Community Sequencing Program to sequence an additional 300,000 cDNA clones [7]. One cDNA microarray (L Hanson, personal communication) and one high density oligonucleotide array [8] have been developed for global gene expression studies. Two large-insert catfish Bacterial Artificial Chromosome (BAC) libraries have been produced: CCBL1 contains a 7-fold genome equivalent of *Hind*III-digested genomic DNA from a 3^rd ^generation gynogenetic female [9], and CHORI-212 contains a 10-fold genome equivalent of *Eco*R1-digested genomic DNA from a diploid male [10]. More than 20,000 BAC end sequences were recently generated from CHORI-212 [11] and 37,251 BAC end sequences have been generated from the CCBL1 library (S. Quiniou, unpublished data). Thus, a physical map of the channel catfish genome is needed to integrate the genetic map with catfish chromosomes, allowing for fine mapping of phenotypic trait alleles such as Quantitative Trait Loci (QTL) and effective positional cloning of genes controlling economically important traits to improve germplasm. The integrated physical map will also be essential for comparative genomic analyses and the assessment of conserved synteny. In addition, a minimal tiling path provided by the physical map would provide the framework for a whole genome sequencing project [12].

To date, most vertebrate physical maps have been produced based on imaging of restriction fragments in agarose gels [13]. Though a proven technique, agarose fingerprinting is time prohibitive with limited personnel, even with the use of band calling software. As a result, alternative fluorescence-based techniques have been developed in order to make use of high-throughput DNA analyzers [14-16]. We report in this study, the construction of a whole-genome BAC-based Fingerprinted Contigs (FPC) map for catfish, *Ictalurus punctatus*, using the 4 color High-Information-Content Fingerprinting (HICF) SNaPshot technique [16]. To our knowledge, this is the first reported assembly of a whole-genome with the SNaPshot HICF in the literature though several other projects are under way (Wheat [17]; Rice [18]). Only two other HICF whole-genome maps have been built, one based on 3 colors fingerprinting for maize [19] and one based on one color fingerprinting for tilapia [20].

## Results and discussion

### BAC fingerprinting

The HICF FPC technique used by Luo *et al*. [16] was chosen as it is well suited to a high-throughput format and the SNaPshot labeling kit is commercially available (Applied Biosystems, Foster city, CA). We fingerprinted 54,816 clones from the *Hind*III CCBL1 catfish BAC library [9]. This library was chosen, rather than CHORI-212, because of the lower DNA sequence polymorphism of the gynogenetic donor. In this library, three percent of the wells were empty and ten percent of the clones did not contain an insert [9]. Also, 1,142 clones were substituted with control clones, so approximately 46,550 clones were effectively fingerprinted. We used the FPMiner v1.2 software (BioinforSoft LLC, Beaverton, OR) to size the DNA fragments from the capillary fingerprinting chromatograms and to identify and remove the clones not meeting our quality standards. Data was then exported to GenoProfiler [21] to remove the plate control wells and clones resulting from potential cross-contamination from the 384 and 96 well plate formats. Altogether, 42,616 fingerprinted BAC clones (91.5%) were analyzed with FPC software, 41,749 were assembled into contigs and 867 remained singletons. The channel catfish genome size is estimated to be 1 Gb [22-24] and the average size of inserts is 165 kb [9], therefore the fingerprinted BAC clones represented approximately a 6.8-fold coverage of the genome.

### Contig assembly

Table 1 summarizes the data for the physical map. The assembly resulted in 1782 contigs with 867 singletons. The resulting contigs of the channel catfish physical map can be accessed at the ARS-USDA-CGRU-Genomics website [25]. Each genome-fold coverage of fingerprinted BACs contributed nicely to the increase in average contig length and physical length of the map (Table 1). After assembly of approximately 7 genome-fold coverage of BAC clones, the contigs reached an average length of 524 kb and the physical map reached a physical length of 0.93 Gb. FPC identified 3,060 questionable (Q) clones in this assembly corresponding to 7.3% of the clones. This is similar to the 11% reported in the maize study [19]. While these numbers are high compared to numbers obtained using an agarose based technique, Q clones in HICF projects can arise from the lower overall reproducibility of the fingerprints hindering alignments of such clones in the assembly. For this project the average fingerprinting reproducibility of the control clones was about 85%. Similarly, Nelson *et al*. [19] showed a reproducibility rate of 75%. As such, the number of Q clones is then less an indicator of the assembly quality in HICF than in agarose fingerprinting. With our current assembly, more fingerprinting of the same library would not likely close the gaps efficiently as we are approaching full-length coverage and because some regions of the genome could be poorly represented in the *Hind*III CCBL1 library. Addition of data from a 1 to 2-fold coverage of the *EcoR*I CHORI-212 library would be more useful but still potentially biased because it was also generated by restriction enzyme digestion. The most effective would be data from a randomly sheared BAC library generated from the same gynogenetic fish used to make the CCBL1 library.

### Contig validation

Three different approaches were used to validate the contig assembly. First, 19 of the largest contigs were anchored to the microsatellite-based genetic linkage map [2] using markers developed from the two most terminal BACs in each contig (Table 2 and Table 3). If no polymorphic marker was available for an end clone, markers were developed from internal BACs which assembled at a Sulston score higher than 1e-40 as these could potentially be weaker points of the assembly. Marker pairs developed within each contig displayed close genetic linkage for 18 of 19 contigs, and 16 contigs were placed on the catfish genetic map. The two sets of markers for contigs 23 and 321 were closely linked within each respective contig (Table 3). However, these markers were not linked to any other marker on the current genetic map (Table 2). One possible explanation is that these two contigs are telomeric and placement of these contigs on the genetic map will require additional linked markers. Contig 84 was found to be the result of a false join (Table 3). Two of the markers present in contig 84 belonged to linkage group U22 and 5 markers belonged to linkage group U16 (Table 2). If this contig represented a junction of the two linkage groups, one would expect the markers on U16 to be linked to the markers on U22, but this was not supported by the linkage analysis. Also, it was unlikely this result was due to a chimeric BAC clone due to the number of clones covering the region between the markers. In the current analysis, the ratio of average contig length to average recombination distance between the most distal markers was 311 kb/cM, compared with 447 kb/cM estimated from the low resolution catfish genetic linkage map [2]. Addition of markers to the genetic map will likely increase map length and decrease the latter estimate. While the ratio of physical to linkage distances varied within the sampled contigs (Table 3), this variability was also evident in the human genome [26]. Further integration of the linkage and physical maps will help clarify this relationship in the catfish genome. This limited analysis produced a contig assembling error estimated at 5%. Similarly, 4% of false joins were found when the maize genome was assembled using the 3 color fingerprinting HICF FPC technique [19]. While the rate of misassembly between two clones should be constant, the probably of a contig containing a false join should be proportional to the number of clones in the contig. Since our analysis was skewed toward the largest contigs, containing 89 to 156 clones, the whole map error rate may be lower. The current validation does not exclude that a few BAC clones could be placed in the wrong contig. Nevertheless the successful anchoring 18 of the largest contigs to the microsatellite linkage map attests to the overall reliability of the contigs and the physical map.

**Figure 1:**
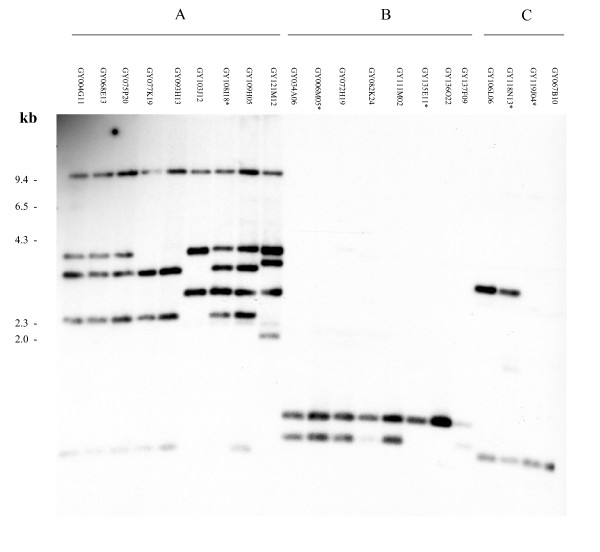
**Southern blot analyses showing MHC class I α3 patterns of 21 BAC clones picked from the CCBL1 library**. A, B and C represent the grouping of the BAC clones according to their restriction patterns and shared bands. * Clones not included in the fingerprinting assembly.

As a second validation, the grouping of the Major Histocompatibility Complex (MHC) class I multigene family was examined to determine if clones containing these genes would be correctly assembled using this HICF technique. MHC class I genes were chosen as the extensive multigenicity of MHC I genes in channel catfish has been shown [27,28]. Twenty-one of the clones previously identified by PCR screening of the CCBL1 BAC library with a MHC class I alpha 3 domain primer pair were examined [28]. Those clones were then grouped either by capillary fingerprinting or by Southern blot pattern following a *Dra *I digest and hybridization with a MHC class I alpha 3 domain probe. Figure 1 shows the Southern blot pattern obtained for each clone. The restriction patterns were segregated into groups A, B, or C. Clones within each group shared one or more bands (Figure 1). Each common band within the group presumably represented a shared MHC I gene between overlapping BACs. Depending on the extent of their overlap, BAC clones shared different bands or even had some unique bands within a group depending on their location in the genome. The remaining 16 BAC clones grouped consistently with the observed Southern blot patterns: A, B, and C corresponded to Contigs 646, 1499 and 1648, respectively, thus confirming the correct assembly of those BAC clones in contigs. Five MHC class I BAC clones were eliminated from the assembly during the quality check of the fingerprinting as they had either too few or too many bands to fit our standards. However, a separate assembly that included these lower quality clones showed that they still mapped according to their grouping in the Southern blot analysis.

Third, the validity of the assembly was also confirmed by the ongoing sequencing of the channel catfish immunoglobulin heavy chain locus [29,30]. Two structurally related genomic clusters of catfish immunoglobulin heavy chain constant region gene segments are known and the distance between the two has been estimated at 725 kb [31]. Six BAC clones representing the two clusters were identified [30]. Those clones from the non-arrayed CCBL2 library were independently fingerprinted, and were localized to two contigs (Contigs 499 and 528, [30]). One clone from each contig/cluster was selected for sequencing and adjacent clones were identified for sequencing using the minimum tiling path and BAC end sequence markers. No discrepancies were found in the assembly of those two contigs when sequencing the clones. Even though FPC could not join those two contigs because the overlap was too small, sequence analysis demonstrated one of the clones from CCBL2 in Contig 499 overlapped with clones GY079K15 and GY099L11 in Contig 528 by 7 kb and 28 kb, respectively (data not shown).

## Conclusion

This is the first whole-genome physical map of the channel catfish. By adapting the SNaPshot based technique to a high-throughput DNA analyzer, we were able to fingerprint a whole genome in a relatively short period of time with limited human resources (two people processed four 384-well plates per day). The resulting stage 1 map was assembled with an initial Sulston score of 1e-40 to maintain a conservative core of contigs as the backbone for further analysis. The validation studies confirmed the reliability of this assembly. We are currently integrating this physical map with our other catfish genomic resources most notably the genetic map [2], to enable QTL studies, comparative genomic analyses and fine mapping of genes to assist in identification of markers associated with economically important traits such as such as disease resistance, growth rates and carcass yield.

## Methods

### BAC library fingerprinting

BAC clones were obtained from the CCBL1 BAC library [9]. The CCBL1 library was produced from a female channel catfish obtained by meiotic gynogenesis. The BAC clones from each 384-well plate were inoculated using the GeneTAC G^3 ^robot (Genomic Solutions, Ann Harbor, MI) in four 96-well 2.2 mL plates (ABgene, Rochester, NY) containing 1.5 ml of LB/12.5 μg/mL chloramphenicol. The clones were grown at 37°C in a HiGro shaker (Genomic Solutions) for 24 hours. Wells E7 and H12 were inoculated with the same BAC clone on all plates to serve as internal controls for plate orientation and fingerprinting quality. The DNA was isolated via an alkaline lysis method with Qiagen reagents (Qiagen, Inc., Valencia, CA) in a 96-well format using an Apricot pipettor (PerkinElmer Life and Analytical Sciences, Wellesley, MA). The DNA was then resuspended in 30 μl ddH_2_O. All steps of the fingerprinting were performed according to Luo *et al*. [16] except that all amounts of reagents including the DNA were divided by 3. Briefly, 12 ul of DNA (approximately 400 ng of DNA) was digested with *Hae*III, *EcoR*I, *Xba*I, *Xho*I, *BamH*I (New England Biolabs, Ipswich, MA) in the presence of 0.1% β-mercaptoethanol and RNase DNase-free (Roche Applied Science, Indianapolis, IN) for 3 hours at 37°C in a PTC-200 thermal cycler (MJ Research, Watertown, MA). Fragments were labeled with the SNaPshot kit (Applied Biosystems, Foster City, CA) at 65°C for 60 minutes C in a PTC-200 thermal cycler (MJ Research). The resulting labeled fragments were precipitated with sodium acetate and ethanol.

### Capillary electrophoresis

To attain high-throughput, the samples were run on a 3730 *xl *DNA Analyzer (Applied Biosystems, Foster City, CA). The fluorescent BAC fingerprinting fragments were resuspended in 10 μl per well of Hi-Di formamide (Applied Biosystems) solution containing 0.05 μl GeneScan-500 LIZ size standard (Applied Biosystems) for at least 1 hour at 4°C. Samples were denatured for 5 min at 95°C, cooled to 4°C on ice and centrifuged (3,220 × g for 2 min) to the bottoms of the microplate wells. The DNA fragments were injected on a 50-cm length, 96 capillary array filled with POP-7 (Performance Optimized Polymer, Applied Biosystems) and resolved using the instrument run module settings shown in Table 4. Initially, the run module and spectral calibration protocols in Data Collection version 1.0 were adapted from sequencing and 36-cm fragment analysis protocols to enable separation and detection of the HICF fragments on the 3730 *xl *DNA Analyzer. However, functionality for both 50-cm fragment analysis and custom 5-color spectral access were enabled by Applied Biosystems in Data Collection versions 2.0 and 3.0. Injection voltage and time were adjusted to optimize fluorescent peak heights and minimize cross-talk between adjacent capillaries.

### BAC contig assembly

The chromatogram files were analyzed with FPMiner v1.2 software (BioinforSoft LLC, Beaverton, OR) to size the fragment and determine fingerprint quality. All samples with fewer than 50 fragments and more than 160 fragments were removed, as were all samples with a Size Standard Matching Quality Score below 0.9 or Fingerprint Editing Quality Score below 10. All fragments present in more than 20% of the samples, which included the vector fragments and potential repetitive DNA, were also removed. Those values were determined after initial examination of the raw fingerprinting data. For channel catfish, the average number of blue (*BamH1*) and red (*XhoI*) fragments per clone were lower than the number of green (*EcoRI*) and yellow (*XbaI*) fragments. As a result clones with unusual ratios of band numbers between the four colors (too high or too low compared to the average number per color) were manually removed. The sample files were then exported to GenoProfiler software [21] to remove the controls and samples demonstrating potential contamination from neighboring wells in the 96- or 384-well plate format. Contigs were assembled from bands between 50–500 bp using FPC software Version 8.5 [32,33]. FPC parameters were adjusted as described by Luo *et al*. [16] and Nelson [19] for the HICF technique. Briefly, as FPC did not accept color labels or fractional sizes, every size was multiplied by 10 and the color labels were converted to non-overlapping numeric ranges by adding a different offset value for each color. As a result, the gel length was set at 18,000 bp and tolerance was set at 4 to obtain the 0.4 bp optimal tolerance value determined by Luo *et al*. [16] for HICF-SNaPshot fingerprinting. The clones had an average of 93 bands and an average size of 165 kb [9]; hence, the estimated band size was set at 1,800 bp in the configuration file. Those values allowed the FPC software to estimate contig length and physical map length. An initial Sulston score of 1e-40 was determined to be optimal for our data set in order to minimize number of contigs without overly increasing the number of clones with questionable alignments (Q clones). Contigs with more than 10 Q clones were reassembled with a stricter cutoff by setting the value of the DQer function of FPC to 10 and setting the step value to 5. We also set the "Best of" function to 50 builds as this setting controlled how many different attempts FPC makes when building the consensus band (CB) maps to try finding CB maps with fewer Q clones. Next, the "Ends to Ends" auto merge function was used with default settings and the stringency was decreased from 1e-40 to 1e-15. To finish, the 'Singles to Ends' function was used with a minimum of 2 ends matching. In that case the stringency was decreased from 1e-40 to only 1e-25 because singletons only had to match on one side. Reproducibility of the fingerprinting technique was assessed by determining the average percentage of shared bands between one selected representative control clone and 100 randomly selected control clones using Genoprofiler.

### Linkage analysis

The terminal BAC clones of each contig, or clones at potentially weak points (resulting from "Ends-to-Ends" assembly) were chosen for linkage mapping. Parents and 48 offspring from two reference families were genotyped, and two-point linkage analysis was performed as described [2]. BAC-specific microsatellites were identified by STRAP sequencing [34] or from BAC end sequencing data (Table 2). Sense-strand primers were synthesized containing a 5' FAM or HEX fluorescent label (InVitrogen Corp., Carlsbad, CA), and anti-sense strand primers were unlabeled (Integrated DNA Technologies, Coralville, IA). The 10 μl PCR reactions were performed using Titanium Taq DNA polymerase following the manufacturer's protocol (BD Biosciences, San Jose, CA) with the following modifications: we used Promega (Madison, WI) dNTPs and added 3 pmol of each primer. The reaction profile was 95°C for 3 min; 2 cycles of 95°C for 1 min and 60°C for 1 min; then 29 cycles of 95°C for 30 s, 60°C for 30 s, 68°C for 30 s, then final extension at 68°C for 4 min in PTC-200 thermal cycler (MJ Research, Watertown, MA). Fragments were separated on a 3730 *xl *DNA Analyzer and sized with GeneMapper v3.0 software (Applied Biosystems).

### Southern blot analysis

The BAC DNA (50 ng) was digested to completion with *DraI*, separated on 1% agarose gels and transferred by capillary action onto Hybond-N+ membranes (Amersham Pharmacia Biotech, Arlington Heights, IL, USA) using standard techniques. Hybridizations were performed in Rapid-hyb buffer (Amersham Pharmacia Biotech) at 65°C and membranes were washed at high stringency (70°C with 0.1× saline-sodium citrate, 0.1% sodium dodecyl sulfate). A MHC class I α3 domain specific probe was amplified by PCR using iProof HF DNA polymerase (Biorad, Hercules, CA) according to the manufacturer's recommended protocol with the following primers: 5'-CAGGTGTAGGTGTGTTTCTG-3' and 5'-GCTACAGGTTTCTTCCCC-3'. The reaction profile was: 1 min 98°C, followed by 44 cycles of 98°C 10 s, 55°C 30 s, 72°C 30 sec, then extension at 72°C for 5 min. Probes were random primed labeled with [32P] 2'-deoxycytidine 5'-triphosphate using a Megaprime labeling kit (Amersham Pharmacia Biotech).

## Authors' contributions

SQ conceived the project, developed the protocols for DNA extraction and fingerprinting on the 3730 *xl *Analyzer, obtained the fingerprints, assembled the physical map, verified contig assembly and wrote the manuscript draft. SQ and GW developed markers for contig verification. GW performed linkage analysis and edited the manuscript. MVD adapted Applied Biosystems Data Collection version 1.0 run modules and spectral calibration protocols to enable separation and detection of the HICF fragments on the 3730 *xl *DNA Analyzer. All authors read and approved the final manuscript.

## Figures and Tables

**Table 1:** Summary of the channel catfish physical map produced from the fingerprinting of 42,616 BAC clones

Genome fold coverage	1X	2X	3X	4X	5X	6X	7X
Physical Length (kb)	303,123	545,178	659,806	732,463	764,469	839,489	930,520
Contigs	1,296	2,024	2,190	2,034	1,775	1,870	1,782
2 clones	599	546	466	284	174	181	158
3 – 9 clones	688	1,266	1,208	930	647	599	466
10 – 24 clones	9	201	442	618	577	632	553
25 – 49 clones	0	11	72	169	277	308	385
50 – 99 clones	0	0	2	33	92	136	184
100 – 199 clones	0	0	0	0	8	14	32
							
Singletons	1,471	1,165	1,119	788	571	751	867

Avg. Contig Length (kb)	233	268	300	359	430	448	521
Longest Contig (kb)	1,011	1,323	1,270	2,124	2,241	2,284	2,651

**Table 2:** Development and mapping of microsatellite markers for catfish BAC clones

Clone	LG^1^	Contig	Successful STRAP	Observed repeat	Sense/Antisense primers^2^	Alleles in mapping reference families (bp)	I.M.^3^
GY001C07	U16	224	(AAT)_10_T	(TAA)_10_	FAM-CCGTGAAGATTTGTGAGACCG/TGGCTGATCTGGAAAGGGAG	239, 242, 251, 254	92
GY003I06	U16	84	(AAT)_9_AAA	unknown	HEX-TTCTTGCCTTAAATCACACATCAG/TGTAACCCAATAGCTTATGTAATGTAC	247, 259, 268, 272	89
GY006O05	U13	2069	(AAT)_9_AAA	(TAA)_11_	FAM-ATAATCGTAATGTCTCTCATCTACTG AATACAGTGAAGCAGAGGGCA	230, 233, 242	47
GY014A20	U10	375	(AAT)_9_AAA	(TAA)_14_	FAM-TCCGTCCTTAACTTTAGCATTTAG/TCAAATGGGAAAATCAAGCGTCG	229, 232, 238, 241, 244	144
GY014G05	U26	191	(AAT)_9_AAA	(TAA)_13+_	FAM-GGATTATTCATGTATAGGGATGCC/CCAGACTCCAATAACCACCATAG	214, 220, 224, 231, 239	138
GY016J21	U14	2122	(AAT)_9_AAAA	(TAA)_9_	FAM-ATGTAAGCTATGTTTCACTGTTGCG/TTTGTCCTGCATTGTAGCCTG	112, 115	48
GY016M05	U26	191	(AAT)_9_AAC	(TAA)_7_	FAM-AATGGCGTTGTTGGAATGGTTG/TTCAACGGAGTCATGTTTGGG	225, 228, 237	90
GY020C24	U16	84	(ATA)_10_G	(TAA)_13+_	FAM-CCTGGTGATAACAATACATAAAGTG/GAAAAGGATAATCAGTATAGAAGGTG	157, 172, 178, 187	48
GY031O20	U22	104	(AAT)_9_AAA	(TAA)_13_	FAM-CATCAGATGGGCATCAGACATG/TAATGAGAAGCGGTGGATCAG	314, 316, 319, 329, 332	189
GY034A19	-^4^	23	(AAT)_9_AAA	(TAA)_7+_	HEX-TTGACTCTAAAAGTGGAACTGTGAG/AAGCCCAGTTTGTAGACATTTTACTC	232, 235, 238	95
GY048N07	U22	84	(AAT)_9_AAA	(TAA)_10_	FAM-CCATTCAGTTAGTCAGTAAAGCTG/CAAAGTAAGGCTTCTGTATTAGTAAC	256, 265, 268, 276, 296, 304	190
GY061E20	U28	220	(AAT)_10_T	(TAA)_8_	FAM-TGGTAAATATGAGAAAAGTAGGCTG/CTTATGGCTGCGGTCGCTTC	185, 214, 217	144
GY063K13	U17	358	(AAT)_10_G	(TAA)_13+_	FAM-ACAAGTATGACTCTGTGCAAATG/GAGATATTAAACGTTACACAGGAAC	418, 424, 427, 431	95
GY065J11	U16	84	(AAT)_10_G	(CAA)_10_(TAA)_15_	HEX-CACGCGCACTCCAAAACAGG/GGTAGTTTCCTAATCTCTTTAAGTC	295, 307, 313, 323, 329	95
GY069G22	U04	422	(AAT)_10_T	(TAA)_16_	HEX-AGGATGTTTCTGCTTCTGATGTG/CATGACTGCCCTACATTTCCTG	361, 364, 370, 373, 376	180
GY074J11	U22	249	(AAT)_9_AAA	(TAA)_14_	HEX-GCTACCCAACAGGCCCAATG/CCAGTCCTGTGTGCATGAAC	365, 368, 371, 377, 384	186
GY082C02	U09	150	(AAT)_9_AAG	(TAA)_14_	HEX-CTGTTAAATATAAAGCTCAGAAATGTTGC/AAGGCGGAGACATTAGCAAAC	322, 324, 334	184
GY082H04	-^4^	321	(AAT)_10_T	(TAA)_14_	FAM-ATAATGCATCACCTGTCGAACCC/TCTCTGTGACATGGCAAGCTG	200, 203, 208	96
GY085J18	U05	158	(AAT)_9_AAG	(TAA)_10_	FAM-CGAAGCTGATCTAAACCAGACG/CTTCTGCTTGTCCGCTTCTTC	234, 236, 242, 251	192
GY088E13	U16	84	(AAT)_9_AAC	(TAA)_15_	HEX-GGATGAACCGCTACAGGAAG/GCCTGTTGCAATACTGATCTGTG	279, 285, 288, 303, 312	90
GY097F03	U14	2122	(AAT)_9_AAA	(TAA)_11_	FAM-CACATTATTGATTATGCACATCGTAC/CTGTTAACTGTTCCATTGACGAG	279, 288, 293, (null)	67
GY098F03	U16	224	(AAT)_9_AAC	(TAA)_11_	HEX-CATAGCAATTTAATGTAACTGACTTAG/ACTCCTCATCTTAGTAATGGTCTG	288, 231, 234, 243	46
GY105F13	U22	249	(AAT)_9_AAA	(TAA)_16_	FAM-CATCATTGGTCTCAACTACAGG/GATGGAGGGTGTAGAAGCAG	282, 291, 294, 297, 300	143
GY105H22	U05	43	(AAT)_9_AAA	(TAA)_15_	HEX-GATTCTTTATATTGTTGCACCTGACG/CTGAACTCAGGGAAGCCCAG	309, 312, 315, 321	136
GY106D19	U23	58	(AAT)_9_AAAA	(TAA)_10_	FAM-GCTGGAGCTGTAATGTCTCTG/TTCTTTTGAAGCCACATAGTAACACAG	176, 179, 182, 185, 188, 209, 212	190
GY107P06	U23	58	(AAT)_9_AAA	(TAA)_11_	FAM-AGTACGCCCACTATCACCAG/GCGTTGAAATGTTGCATTCTGTG	230, 242, 245, 248, 251	95
GY110K13	U22	104	(AAT)_9_AAA	(TAA)_10_	FAM-ACATTACCCGAAGGTCAGTTTG/TACCCACTGCCATCCCTTATC	225, 231, 234, 237	188
GY112F18	U16	84	(AAT)_9_AAC	(TAA)_13_(CAA)_3_	FAM-CGGATAAGAAGCTGTTGCTAC/GTAATCATTGGCTTGGTAATTATTAGAC	300, 302, 319, 328	95
GY113C07	U05	43	(AAT)_9_AAG	Unknown	FAM-GTATGACACGACATCCAAAACGG/GCCTGACTTTCTGGAATTTTCTTG	119, 122	142
GY113J02	U10	375	(AAT)_9_AAAA	(TAA)_17_	HEX-CACGTTCAGGCCAATACAACAC/TGAAGTGGTATCATATAACATTAGGC	271, 301, 313, 319	94
GY115E15	-^4^	321	(AAT)_10_G	(TAA)_16_	FAM-ACATAGGATTGTAGGAAAACTAACTG/AGTGATTAATCTATGAATTCATGCTG	215, 219, 223	94
GY116H16	U09	150	(AAT)_9_AAA	(TAA)_13_	HEX-TTGTGAGTGTTCTTCATGTGCTG/CCATATCAGATTGATGCATCCTTAC	187, 193, 196, 199, 208	131
GY119G01	U05	158	(AAT)_9_AAA	(TAA)_18_	FAM-CGTGCAACAGAATATTCATTAAATGTG/GTTACTGTATTAATTTCACCCTTGTGG	236, 253, 259, 262, 265	190
GY121F03	-^4^	23	(AAT)_9_AAA	(TAA)_12_(CTA)_4 _(CAA)_8_(TAA)_7_	HEX-TATCAGTGGGCATGCACATCTG/GATGTTATTAACTGATCATGCTAAATTATTG	323, 341	46
GY124F07	U27	1994	(AAT)_9_AAA	(TAA)_9_	HEX-CGTTATCAAATACAGTGGTGCTTG/GTTTATATTTGTAACACTCAGTTTGTGG	278, 284, 290, 293	47
GY125G21	U27	1994	(AAT)_9_AAA	(TAA)_11_	HEX-AATTCTGTATGCATCTCGGAAATAGC/CTCTTCTGGAAACTCTTACAGTG	210, 219, 225, 231, 234	86
GY127H06	U28	220	(AAT)_9_AAA	(TAA)_23_	HEX-CTTCTTTGCACGGATCACCTG/TTCATTCCCCTCATCACACAG	257, 263, 269, 281	92
GY132P10	U17	358	(AAT)_10_G	(TAA)_11_	FAM-GGGCAGTGGTAGCTTAGTGG/CAGGGAAGCTCAGACTTTTGG	239, 249, 258, 261, 264, 267	184
GY135E18	U14	13	(ATA)_10_AGC	(TAA)_6_	HEX-CAAAATGAACAATCAGGGTAAAGGC/TGGATTACTACACTTTCATATGTGG	331, 343, 355, 363	140
GY137B18	U16	84	(AAT)_9_AAA	(TAA)_16_	HEX-CCTGGTATGACCCGTTTCTTG/TGCGCGAGACACATTTATACC	169, 181, 190, 192, 195	92
GY137K09	U22	84	(AAT)_9_AAA	(CAA)_3_(TAA)_10_	HEX-CCTATACTCTGCCATTCAGTTAG/TGACCATCCCAGTACAAATGC	132, 143, 146, 148, 169	139
GY138C24	U13	2069	(AAT)_10_G	(TAA)_12_	FAM-CATAGCTGTAGCAGGCAGATTG/ATACGTTTGTACTTGTTGCTAGTGG	293, 296	94
GY143D16	U04	422	(AAT)_9_AAAA	(TAA)_12+_	HEX-GCGTCTGATGAGAAACAAACTATG/ACCAGGAAATTCACCCAAGAC	142, 145, 155	135
GY144N04	U14	13	(AAT)_9_AAAA	(TAA)_10_	FAM-CTTCCTCAGACTGGTGTCAC/TCTACACACCTTCGGTGCTTG	226, 232, 235, (null)	47

^1 ^LG = linkage group

^2 ^Sense-strand primer was 5' labeled with FAM or HEX fluorophore.

^3 ^Informative meioses

^4^No linkage to markers on the current genetic map

**Table 3:** Verification of physical map integrity by linkage mapping of clones from each end of the contig

Contig	Number of Clones	Length (kb)	Left clone	Right clone	Most distal internal clone	r^1^	LOD^2^	Linkage Group
Contigs with markers on terminal BAC clones								
13	93	848	GY144N04	GY135E18	not used	0.09	7.91	U14
23	138	1136	GY121F03	GY034A19	not used	0.02	11.45	Unknown^3^
43	121	807	GY105H22	GY113C07	not used	0.01	29.49	U05
58	106	753	GY107P06	GY106D19	not used	0.01	25.59	U23
104	99	767	GY110K13	GY031O20	not used	0.01	49.69	U22
150	128	1233	GY116H16	GY082C02	not used	0.03	19.63	U09
158	96	816	GY085J18	GY119G01	not used	0.01	51.17	U05
220	130	1429	GY061E20	GY127H06	not used	0.09	15.29	U28
321	91	599	GY082H04	GY115E15	not used	0.04	20.51	Unknown^3^
358	96	1068	GY063K13	GY132P10	not used	0.01	24.10	U17
375	103	1006	GY014A20	GY113J02	not used	0.06	18.00	U10
422	92	816	GY143D16	GY069G22	not used	0.02	31.16	U04
1994	120	1070	GY124F07	GY125G21	not used	0.02	10.58	U27

Contigs with markers on one terminal BAC clone								
191	89	694	GY016M05	n/a	GY014G05*	0.05	9.41	U26
224	129	1180	n/a	GY001C07	GY098F03*	0.00	13.55	U16
249	156	1064	GY105F13	n/a	GY074J11*	0.01	38.07	U22
2069	128	1034	n/a	GY006O05	GY138C24*	0.00	13.55	U13
2122	106	409	n/a	GY016J21	GY097F03*	0.00	13.85	U14

Mis-joined contig								
84a	139	1402	GY137K09	n/a	GY048N07*	0.00	40.34	U22
84b	139	1402	n/a	GY137B18	GY020C24*	0.00	13.25	U16

^1 ^Percent recombination based on two-point linkage

^2 ^Log_10 _likelihood ratio of linkage vs. non-linkage

^3 ^No linkage to markers on the current genetic map

* When a polymorphic marker was not available (n/a) for a terminal BAC clone, this most distal mapped clone was used for linkage analysis.

**Table 4:** ABI 3730 XL run module for SNaPshot HICF 4-color fingerprinting using POP-7 polymer in a 50 cm capillary

Setting	Value
Oven Temperature	63
Buffer Temperature	35
PreRun Voltage	15.0
PreRun Time	180
Injection Voltage	2.0
Injection Time	20
First ReadOut Time	200
Second ReadOut Time	200
Run Voltage	15.0
Voltage Number Of Steps	10
Voltage Step Interval	20
Voltage Tolerance	0.6
Current Stability	30.0
Ramp Delay	200
Data Delay	400
Run Time	2100
